# Modeling security evaluation framework for IoHT-driven systems using integrated decision-making methodology

**DOI:** 10.1038/s41598-024-62066-3

**Published:** 2024-05-28

**Authors:** Habib Ullah Khan, Yasir Ali

**Affiliations:** 1 Accounting and Information Systems, College of Business and Economics, Qatar University, Doha, Qatar; 2 Shahzeb Shaheed Government Degree College Razzar, Swabi, Higher Education Department, Peshawar Khyber Pakhtunkhwa, Pakistan

**Keywords:** GTM, TOPSIS, IoHT assessment framework, Authentication security requirements, Computational models, Data processing

## Abstract

The intensification of the Internet of Health Things devices created security concerns due to the limitations of these devices and the nature of the healthcare data. While dealing with the security challenges, several authentication schemes, protocols, processes, and standards have been adopted. Consequently, making the right decision regarding the installation of a secure authentication solution or procedure becomes tricky and challenging due to the large number of security protocols, complexity, and lack of understanding. The major objective of this study is to propose an IoHT-based assessment framework for evaluating and prioritizing authentication schemes in the healthcare domain. Initially, in the proposed work, the security issues related to authentication are collected from the literature and consulting experts’ groups. In the second step, features of various authentication schemes are collected under the supervision of an Internet of Things security expert using the Delphi approach. The collected features are used to design suitable criteria for assessment and then Graph Theory and Matrix approach applies for the evaluation of authentication alternatives. Finally, the proposed framework is tested and validated to ensure the results are consistent and accurate by using other multi-criteria decision-making methods. The framework produces promising results such as 93%, 94%, and 95% for precision, accuracy, and recall, respectively in comparison to the existing approaches in this area. The proposed framework can be picked as a guideline by healthcare security experts and stakeholders for the evaluation and decision-making related to authentication issues in IoHT systems

## Introduction

The current healthcare systems have been operationally supported by the application of many emerging technologies. In the list of emerging technologies, the Internet of Health Things (IoHT) has provided industrial and technical solutions to cope with emerging challenges in the healthcare department. IoHT significantly altered the healthcare environment by enabling accurate and timely processing of patient data through real-time monitoring. Apart from, offering a plethora of healthcare services, smart healthcare IoHT devices have been adopted to provide healthcare services including early detection of infectious illnesses and real-time health monitoring^1^. IoT platforms enable doctors to consult and treat patients and manage their records well^2^. Modern healthcare systems are composed of various IoHT devices that use various actuators and sensors during the transmission and receiving process of patients ‘sensitive data. IoT also helped in augmenting the healthcare system by reducing the different costs related to hospital visits, transportation, and human resources^3^. IoT devices work like add-ons to make IoHT systems smarter, better, and easier to use but still, there are some serious security and privacy issues affiliated with their application that require addressing. IoHT devices are susceptible to different attacks for several reasons physical attacks on unattended components are easy, wireless in nature, and low capabilities and resources^4^. The effects of these problems become more adverse in a health environment particularly due to the handling of very sensitive data due to the reasons that patients never want to disclose or compromise of their identity or data by any intruder or eavesdropper. Therefore, data handled in healthcare is required to be protected from intruders or hackers as the entrance of malicious or unauthorized user entry will not only jeopardize the data but will also lead to the compromising of entire network resources and infrastructure. IoHT devices lack security and suffer from authentication and cyber security issues that need to be properly checked for identity as any intruder will lead to the security of the entire network.

The authenticity/identity management of IoT devices deployed in the healthcare industry is very critical as the quantity and complexity of IoT devices in this setting are rapidly growing. Similarly, the majority of IoHT devices are susceptible to a range of cyber threats and assaults. IoT applications and data are also sensitive, so it is essential to assess and implement the most appropriate authentication technique for the safe authentication of IoHT devices. In a similar vein, technological advances are causing an exponential increase in the number of authentication methods. The protection of IoT devices, particularly in the health department has become a pressing concern during the past ten years and it has attracted the attention of many researchers to pursue research in this domain. For this purpose, an array of security methods, processes, models, frameworks, and schemes have been suggested to provide tentative solutions to security-related problems over the last few years in the health department. This trivial intensification of security authentication solutions has led to many decision-making issues and uncertain situations for the different people working in the healthcare domain. The assessment and decision-making regarding the selection and installing the most appropriate authentication technique or solution is also a major concern and challenging task for IoT network managers and decision-makers. This is the reason that authentication has proven to be the most difficult task in the context of healthcare. Therefore, a smart and intelligent authentication evaluation model is required to evaluate the existing authentication techniques/schemes and to deploy the most suited and rational authentication solution to keep the data protected from outside the world by disallowing illegal entry from the outside world by checking identities of IoT devices in the healthcare domain. The robustness of authentication schemes can be judged by the number of features it embroils for the authentication procedure of IoT-based systems but the importance of the features becomes more viable in medical care environments where sensitive data related to patients is transmitted. These features are not only the building blocks of IoT devices but they also provide network-based security. According to Hamidi et al.^5^, the data security of the internet is defined by three main dimensions such as integration, privacy, and availability. But, in a healthcare environment, security cannot be accomplished by these three dimensions; and more features such as integrity, availability, confidentiality, key agreement, scalability, password change, etc. must be required to achieve full-pledged security.

In this research, a features-based evaluation framework is introduced to deploy a security-preserving authentication solution for IoHT devices. The core theme of this IoHT assessment framework is to evaluate and select the most suitable authentication solution wrapping all the authentication features. The recommended framework identifies the authentication issues based on conducting surveys with healthcare professionals and then it identifies various features from literature and survey-based studies. The feature extraction and selection working procedure is accompanied by the Delphi method. Finally, the criteria of evaluation are designed based on the collected features and consulting with security experts in IoT security. The selected features provide a complete package of security for IoHT devices, and the authentication mechanism selected by the IoHT assessment framework in the healthcare domain is assessed and ranked based on the features. The included features in this study are: forward security, mutual authentication, privacy protection, integrity, key agreement, password change, scalability, confidentiality, and availability. After finalizing the features, the next step is to apply the mathematical approach to evaluate the authentication alternatives. The assessment procedure is conducted using the graph theory and matrix (GTM) approach. The accuracy and consistency of the results are verified and validated by applying the AHP-TOPSIS approach, supported by conducting surveys with security experts' groups in this domain.

This research contributes in the following ways.

- A feature-based assessment framework is presented to overcome the challenges involved during the decision-making process of installing the most ideal authentication scheme in the healthcare environment. This is the first kind of framework of its nature to present a feature-based assessment framework for authentication schemes in the IoT environment. The proposed methodology is supported by multi-methods as it uses a variety of methods, like the GTM approach, which has been applied to the evaluation and quantification of alternatives. The Delphi method has been applied for feature identification and analysis. The integrated methods, such as AHP-TOPSIS, have been applied for the validity and verification of the proposed model. A survey-driven case study has also been conducted to validate and verify the results of the given evaluation framework. The previous assessment frameworks were based on one or two methods. Testing and validating mechanisms are also missing in the existing methods in the current literature.
- This framework evaluates the authentication solution/schemes based on their core security features. It is the first type of work to address the authentication issues of IoT devices in the healthcare environment by taking into account the most important authentication features like mutual authentication, key agreement, forward security, confidentiality, privacy protection, password change, integrity, availability, and scalability. Although many authentication evaluation frameworks have been proposed, the most essential features have not been addressed. The assessment criteria defined with these features cover all aspects of authentication as suggested by the expert’s panel. The selected features were collected from a literature-based study and a comprehensive survey-based study. However, the features or attributes used by previously presented models are only based on literature. Furthermore, a feature analysis is conducted by applying a well-known Delphi method based on conducting extensive questioning and answering sessions.
- The proposed assessment framework uses a novel technique, i.e., graph theory and matrix (GTM), for assessment and decision-making related to authentication solutions in the medical care environment. Whereas, the existing evaluation models are based on traditional decision-making approaches such as AHP, TOPSIS, ANP, etc., which suffer from different limitations in their application. In the literature study, it has come to the observation of the authors that all the decision-driven systems or evaluation models are using the AHP or TOPSIS approaches for security assessment. But these methodologies will be acceptable whenever the features depend upon each other. The AHP method has been applied by several authors but according to Munier et al.^6^, it does not work well where the number of criteria and sub-criteria are many and show complexity. This method also lacks visualization of the interrelationships among the features. The majority of previous evaluation models lack sensitivity analysis and validation. In comparison to the proposed study, all the current methods are based on using old methods in the case of evaluating the authentication solutions. However, the suggested evaluation method presents a new approach to evaluation by supporting both hierarchy and feature visualization. It adopts logical and mathematical procedures for analyzing, evaluating, and making decisions^7^. The proposed evaluation framework removes the evaluation limitations in the currently available methods.

The remaining sections of this paper are organized as: Section "Related work" is about discussing the related work. Section "Methodlogy of the proposed assessment framework" describes the methodology of the proposed IoHT authentication assessment framework. Section "Results and discussion" is related is elaborating the results and discussion, Section "Practical implications" discusses the practical implication of this work, and Section "Conclusion and future work" brings the conclusion of this work.

## Related work

The security evaluation of the IoT-based healthcare system has been a continuous process in the last few years. A comprehensive literature study is conducted to identify the research gaps. Although there are many evaluation models intended for the security of IoT devices in different fields, the central emphasis in the proposed study is to investigate the existing literature only for the evaluation frameworks, models, and methods employed for the IoT-based systems in the healthcare area. These models often use MCDM-driven methods^8–12^ and Artificial intelligent approaches^13,14^ for the assessment purpose. However, the literature study is restricted to highlighting only those research works that are targeted to perform security assessments in healthcare environments using multi-criteria decision-making (MCDM) techniques. In this section, the comparison of the proposed evaluation framework in terms of features and evaluation methods with similar works in the literature is described.

Haghparast et al.^15^ introduced a security-based evaluation framework to provide security solutions within the healthcare system. The authors applied fuzzy-ANP for the evaluation based on using five (5) features such as networking, services, interoperability, privacy, and dependability. This study addresses the security of IoT devices in terms of layers in the healthcare environment.

Al-Zahrani et al.^16^ the study is focused on evaluating the usable security of healthcare technologies by using a unified technique. The evaluation procedure is conducted by using ANP, TOPSIS, and fuzzy logic. The criteria of evaluation are using four (4) different evaluation features. The evaluation features include confidentiality, satisfaction, integrity, and availability.

Zarour et al.^17^ evaluated the effect of the Blockchain models on maintaining the security of electronic health records (EHR). The adopted fuzzy ANP-TOPSIS approach for evaluation for eight alternatives (8) based on six (6) evaluation parameters such as identity, data security, data monitoring, immutability, consensus, and value.

Enaizan et al.^18^ built a decision-driven system for the security and privacy of electronic medical records (EMR). The proposed framework adopts AHP-TOPSIS techniques with the support of K-means clustering to identify the critical factors. This research study covers five (5) different hospitals in Malaysia. Privacy and security evaluation are the main factors used in their study and sub-factors include authentication, integrity, availability, non-repudiation, and unauthorized access.

Algarni et al.^19^ also applied fuzzy AHP-TOPSIS approaches for checking the security level related to the web-based medical image processing systems. They designed the evaluation criteria based on confidentiality, authentication, authorization, availability, integrity, utility, procession, and resilience. The key motivation of this study is to investigate and evaluate the different aspects of MRI devices like Computed Tomography (CT) scans, ultrasound, and X-ray machines based on respective criteria and goals.

Ansari et al.^20^ study is aimed to put forward a quantification model for the assessment and selection of the best security requirement engineering technology in the healthcare environment. The major idea behind their work is to select the best SRE method based on criteria features. The major components of their proposed criteria are security goals, security requirements, threats, risks, assets, vulnerability, stakeholders, and stakeholders.

Kumar et al.^21^ presented a hybrid-based symmetrical methodology based on AHP-TOPSIS approaches for evaluating the factors that are impacting information security in healthcare. According to their study, the major factors that are contributing to healthcare information security are social engineering, malware, and low access control management. human error, outdated information technology infrastructure, and med-jacking.

Ahmad et al.^22^ conducted empirical analysis using computational methodology for choosing the best security technique for healthcare devices. Their study uses AHP, Hesitant Fuzzy, and TOPSIS methods for evaluation by using security features such as encryption, biometrics, authentication, security token, password, access control, backup, software recovery, error detection, and version control.

Huang et al.^23^ applied the ANP method to evaluate the IoHT systems. It combines the different kinds of features from the literature and well-known security standard ISO/IEC 27,002 (ISO 27,002. The main evaluation parameters in this study are confidentiality, availability, authentication, safety, continuity, trustworthiness, auditing, network monitoring, secure key, non-repudiation, and secure key management.

Hussain Seh1 et al.^24^ worked on forwarding an efficient and effective security assessment framework for web-based healthcare applications. The proposed computational model works on two well-known MCDM approaches such as AHP cum TOPSIS. The criteria consisted of features such as authentication, data validation, encryption, limit access, robustness, revoke access, and audit by evaluating ten (10) healthcare web applications. Similarly, In another study, where Kaur et al.^25^ also focused on evaluating the risk of web-based healthcare applications. The authors adopted an adaptive neuro-fuzzy inference system model for prioritizing the risks related to web-based applications in the healthcare field.

The complete details about the different studies in terms of methodological approach, feature selection, healthcare focus, advantages, and disadvantages are given in Table 1.

**Table 1:** Comparison of proposed work with the existing methodologies.

Study	Year	Proposed method	Evaluation parameters	Security focus in healthcare	Pros ( +) | Cons (-) (In comparison to the proposed study)
Haghparast et al.^15^	2021	Fuzzy ANP	Networking, Services, interoperability, privacy, security and dependability	Evaluating the IoT devices for layers in healthcare	( +) Eliminates the problem of hierarchy (-) Prone to human error (-) A limited number of parameters (-) Results validations are missing
Al-Zahrani et al.^16^	2020	Fuzzy ANP-TOPSIS	Confidentiality, Satisfaction, Integrity, and Availability	Assessment of usable security of healthcare software	( +) A multi-methods approach ( +) Efficient methodology (-) Some other security parameters are missing (-) Survey and results validation required
Zarour et al.^17^	2020	Fuzzy ANP-TOPSIS	Identity, data security, data monitoring, immutability, consensus, and value	Evaluation of the impact of Blockchain models on EHR	( +) Sensitivity analysis ( +) Efficient decision-making methods (-) Sensitive to weight assignment (-) Transparency issues (-) Features analysis is missing
Enaizan et al.^18^	2020	AHP-TOPSIS	Authentication, integrity, availability, non-repudiation, and unauthorized access	Decision support system for the security and privacy of electronic medical records (EMR)	( +) Very simple approach ( +) Flexible model (-) Performance degradation with increasing criteria (-) No results validations (-) Limited evaluation parameters
Algarni et al.^19^	2020	FAHP-TOPSIS	Confidentiality, authentication, authorization, availability, integrity, utility, procession, and resilience	Analysis of the level of security of web-based medical image processing systems	( +) Advanced MCDM method application ( +) Eliminating the volatile scale of ranking (-) No validation (-) The limited set of criteria
Ansari et al.^20^	2020	Fuzzy TOPSIS	Asset, security requirements, threats, risks, vulnerability, and stakeholders	Selection of best security requirements engineering technology for the healthcare software development	( +) Effective for the software developers ( +) Simple evaluation methodology (-) Limited set of features (-) Validation mechanism is not mentioned
Kumar et al.^21^	2020	AHP-TOPSIS	Social engineering, malware, and low access control management. human error, outdated information technology infrastructure, and med-jacking	Assessment model for factors affecting the healthcare information security	( +) Effective assessment methodology ( +) Results validation (-) Classical way of data collection (-) Survey’s validity
Ahmad et al.^22^	2022	AHP-TOPSIS	Encryption, biometrics, authentication, security token, password, access control, backup, software recovery, error detection, and version control	Computational Methodology for assessment of healthcare devices	( +) Effective decision-making method ( +) Good comparison with similar studies (-) No features evaluation (-) Classical way of data collection (-) Performance degradation with increasing criteria
Huang et al.^23^	2020	ANP	Confidentiality, availability, authentication, safety, continuity, trustworthiness, auditing, network monitoring, secure key, non-repudiation, and secure key management	Evaluation model for IoMT solutions in the healthcare sector	( +) Simple evaluation method (-) No feature evaluation (-) No validity of survey (-) Lack of platform validation
Hussain et al.^24^	2022	Fuzzy AHP-TOPSIS	Authentication, data validation, encryption, limit access, robustness, revoke access, and audit	Risk assessment of web-based healthcare applications	( +) Advanced method of evaluation ( +) Effective decision-making methods (-) No features evaluation (-) Classical approach to data collection
Kaur et al.^25^	2020	Adaptive Neuro-Fuzzy Inference System	Access control, integrity, confidentiality, availability	Decision-making system for prioritization of web-based healthcare applications	( +) Advanced method of assessment ( +) Extended data collection procedure ( +) Good sample size (-) Limited criteria features (-) The classical method of data collection
Attaallah et al.^26^	2023	Fuzzy AHP-TOPSIS	Confidentiality, Integrity, Availability, access control, Authentication	Evaluating the security risks in healthcare web applications	( +) Simple evaluation method (-) Classical procedure followed by a survey (-) No framework validation (-) No result testing
Obidullah et al.^27^	2024	HF AHP-TOPSIS	Transportation, healthcare and IoT-related risks	Assessment of IoTT device applications in emergency healthcare	( +) Innovate and integrated assessment approach ( +) Comparative analysis (-) Classical method of data collection (-) Some important features are excluded (-) Limited selection of alternatives
Ahmed et al.^28^	2023	Fuzzy AHP	Integrity, Robustness, authentication, confidentiality and complexity	Evaluating the security of digital watermarking techniques for medical image	( +) Simple evaluation approach (-) No comparative analysis (-) Lack of results validation (-) Classical approach to data collection
Ahmed et al.^29^	2023	Neutrosophic AHP	Security, privacy, access control, authentication, integrity, availability, data centers and secure infrastructure	Criteria prioritization for secure and lightweight storage for e-healthcare services	( +) Updated evaluation methods ( +) Simple evaluation approach (-) No comparative analysis (-) No proper data collection procedure (-) Lack of result validation (-) Lack of comparison with similar approaches
Proposed work	2024	Snowballing(Both forward and backward) Delphi, GTM (AHP-TOPSIS)	Confidentiality, password change, Privacy protection, forward security, integrity, scalability, availability, mutual authentication	Integrated decision-making methodology for evaluation of the IoMT systems	( +) Advanced and hybrid assessment methodology ( +) Updated and efficient decision-making methods ( +) Features analysis ( +) Results validation and testing ( +) Comparative analysis with existing approaches ( +) Snowballing for the features selection process ( +) Leveraging the Delphi method for data collection (-) Complexity in integrating multiple approaches

## Methodlogy of the proposed assessment framework

**Figure 1:**
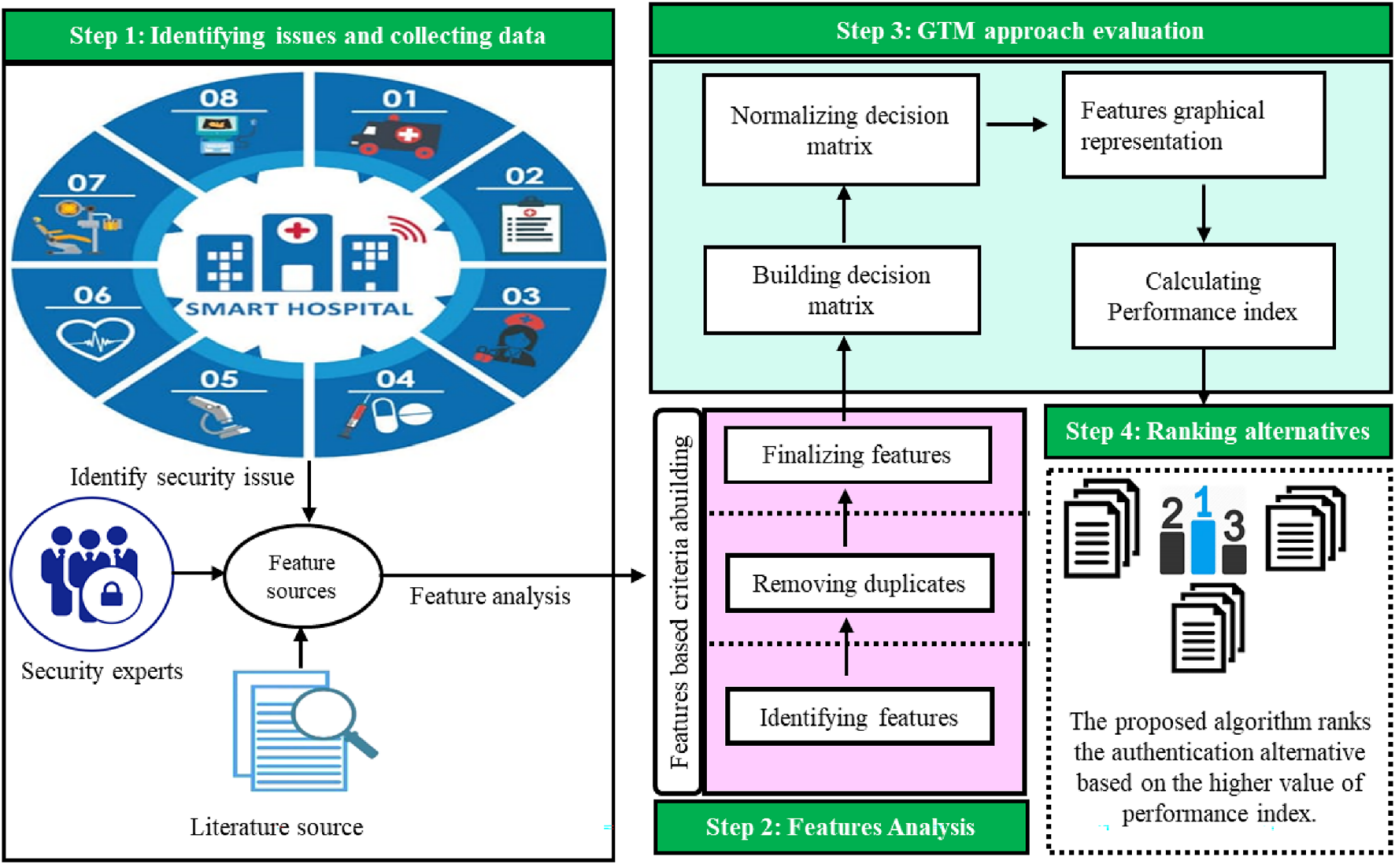
Features-based IoHT authentication assessment framework.

The main objective of this framework is to evaluate the authentication solutions or schemes based on designed criteria which consist of different authentication features. The features of the criteria are intended to provide a holistic security solution, as the IoHT architecture is composed of various layers such as the application layer, support layer, network layer, and perception layer. Security needs to be incorporated at each layer, and this can only be done by considering all the required security attributes of an IoHT-based system. At the very first layer, the perception layer of IoHT architecture, different IoT devices, nodes, and sensors are operating such that they deal with the physical design of the network. The major threats and attacks at this layer are eavesdropping, node capture, malicious and fake nodes, replay, and timing attacks^30^. This is the main target of hackers to utilize or use their sensors. A proper evaluation mechanism is required at this layer to check the devices for security and choose the most secure authentication solution that is to be employed in these sensors, nodes, and IoT devices in the healthcare domain. The selection of a rational authentication scheme for devices in the IoT is very important, as they are used for monitoring and analyzing fragile data related to patients. The devices participating in the network are required to be thoroughly authenticated by using a robust and efficient assessment method. In this research work, the main focus is to evaluate and make decisions about the authentication solution for IoT devices in a healthcare environment by using various features related to authentication. This framework works based on the principle of collecting features from literature, and then these features are used for the selection of feature-based authentication solutions intended for IoHT devices. The central agenda of the suggested framework is to consider the importance of features in authentication and to determine the value of each feature. This mathematical framework provides the foundation for incorporating the features in authentication and helps determine which features are to be included and why they are important for the authentication of IoT devices. The complete structure of the proposed IoHT assessment authentication framework for IoT devices is given in Fig. 1.

The IoHT authentication assessment framework is completed in four different stages. Authentication issues are identified, and data related to the authentication features is collected from an expert panel in the first step. A vigorous and complete case study is conducted to get a deep understanding of the authentication issues and challenges. In the second step, the highlighted issues are analyzed and features are categorized. The features are selected by considering the issues prevailing in the authentication of IoT devices. The complete procedure is depicted in the second step of the recommended assessment framework. In the third step, the GTM approach was applied for the assessment and selection of the best devices based on the collected features, and finally, the ranking was performed by accompanying the mathematical procedure.

In this research work, a case study is performed to understand the authentication issues and to provide solutions in terms of features targeted towards authentication. In the first case study, the challenges and issues related to authentication are identified by the medical personnel, and in the second case, a meeting with the expert in IoT security is arranged to provide solutions to the authenticating issues and challenges based on features. The complete and comprehensive details of all steps involved in the proposed research framework are given below.

### Identifying authentication issues

**Figure 2:**
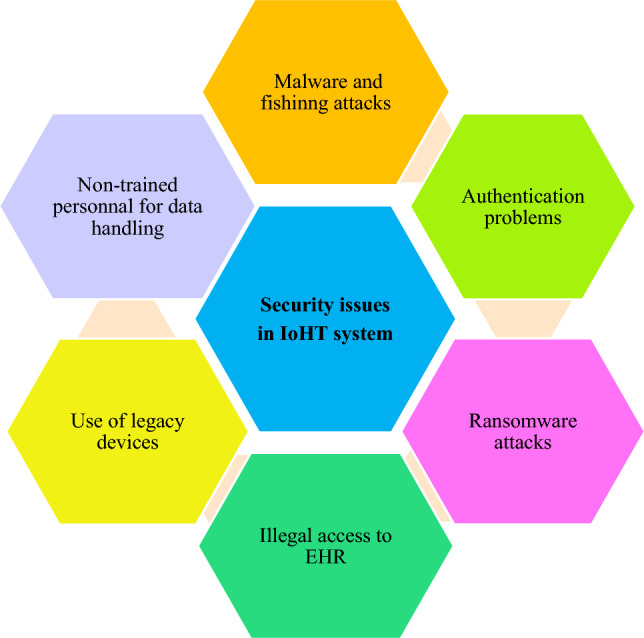
Major security issues in IoHT-based system.

The major purpose of the proposed evaluation model is to identify authentication-related issues and provide a solution based on the development of this model. A comprehensive literature study is conducted to know about the nature of problems existing in the current authentication methods applied to the security of the healthcare system. Among the security challenges, patient authentication is a major concern for healthcare departments^31^. The existing authentication scheme in healthcare suffers from insufficient passwords and secure data storage^32^. Similarly, the anonymity and security against mobile device theft attacks are also not addressed by the existing authentication schemes. For instance, the authentication schemes presented by Chen et al.^33^ provide better authentication but suffer from patient anonymity, stolen mobile device resistance, and impersonation attack resistance. Similarly, the authentication protocol suffers from message authentication, patient anonymity, and stolen mobile device resistance^34^. Chiou et al.^35^ authentication protocol also has the same limitations of stolen mobile device resistance and patient anonymity. Mohit et al.^36^ presented a better security protocol but it lacks the features of non-repudiation. Additionally, medical text data is transmitted over an open communication medium, and it is highly susceptible to security and privacy attacks^37^. According to the literature, many challenges faced by the healthcare system are related to the software's usability as well. After investigating various studies, it is observed that the existing authentication schemes can be improved or a new authentication scheme can be designed by eliminating the existing shortcomings or adding more features to meet all the security requirements. A survey is systematically conducted to identify and highlight the authentication issues in the medical care environment. The staff operating in this area want easy-to-use software security mechanisms. Similarly, the existing authentication schemes employed for the security of IoHT are properly examined to find out the security loopholes. In this regard, open-ended interview questions are asked of the medical personnel in the first phase of the case study to get a deep insight into the authentication problems in the IoHT domain. The responses collected from the expert’s group are analyzed, and a complete catalog is created. From this observation, it comes to light that it is imperative to build an evaluation framework for the selection of authentication schemes due to the lack of understandable and technical knowledge. These issues are divided into different categories and translated into features. Based on the literature and survey, the major security issues prevailing in the IoHT-based system are given in Fig. 2.

### Procedure of selecting features

After identifying issues in the healthcare department related to authentication, the second step is about analyzing and categorizing issues to build feature taxonomies. For this purpose, a case study was conducted to select ten (10) information and network security experts. The identified issues were analyzed, and features were selected based on the security requirements of the medical care environment. The medical IoT network engineers were given security-related questions to deeply understand the nature of authentication problems. The current authentication solutions employed in the literature were also investigated based on the features and limitations of the existing evaluation models. An organized and systematic procedure for the analysis of features is conducted. The feature selection process involves several steps in the first step, features related to authentication are identified based on a literature study and survey. Some features are used by more than one author, so duplication is removed and a final list of features is selected. A questionnaire consisting of forty-four (44) questions is prepared for the collection of data from the medical IT staff working in different hospitals in Pakistan and Qatar. Questions related to authentication issues and their classification into different features are depicted in Table 2. A feature analysis is conducted to learn about the authentication challenges and to reflect on the authentication issues in the authentication method or scheme for future purposes.

**Table 2:** Features-based data collection questions.

Feature: Confidentiality
Q1: How confidentiality is important for the authentication of IoT devices?
Q2: How confidentiality can achieve maximum security related to authentication?
Q3: Rate the role of confidentiality for IoT devices in the healthcare environment
Q4: How does confidentiality add to the security of IoHT?
Feature: Integrity
Q5: Does the integrity is essential for IoHT system?
Q6: Does integrity protect unauthorized access in healthcare?
Q7: How does integrity maintain access to IoHT nodes and servers?
Q8: Do the medical devices exhibit enough integrity of data?
Q9: How integrity is important for security criteria?
Q10: Scale the importance of the integrity of data for IoT devices
Feature: Availability
Q11: What is data availability of data in the healthcare environment?
Q12: Does availability affect the security of IoT devices?
Q13: How does availability provide a shielding effect against DoS/DDoS attacks in IoHT?
Q14: How it is important for security criteria defined by this research?
Q15: What is the impact availability on IoT vertical applications related to the healthcare sector?
Feature: Key agreement
Q16: What will be the impact of a key agreement on authentication in IoHT?
Q17: How session key will affect the authentication?
Q18: How does it add to the security of IoT devices in the healthcare industry?
Q19: What are the current encryption schemes for IoHT devices?
Feature: Password change
Q20: What are the password-based authentication methods employed?
Q21: What are the limitations of using passwords as authentication options?
Q22: Is password-based authentication sufficient to meet the needs of security?
Q23: Do IoHT applications support multi-factor authentication?
Q24: How do IoHT applications authenticate every time they connect?
Q25: Does the password of every IoHT device is unique?
Q26: What is password expiry duration?
Q27: What is the complexity of passwords?
Feature: Scalability
Q28: What is the number of users authenticated by the IoHT application?
Q29: How quickly the number of IoT devices are changing?
Q30: Are the existing techniques enough to satisfy the authentication or not?
Q31: Are the existing authentication methods supporting the scaling up of new devices or applications?
Feature: Mutual authentication
Q32: What are the procedures employed for mutual authentication?
Q33: What are the issues related to mutual authentication?
Q34: Do all the devices are mutually authenticated with other devices?
Q35: What are existing mutual authentication schemes?
Feature: Privacy protection
Q36: What is the level of privacy in the healthcare environment for existing IoT applications?
Q37: Do the IoHT applications provide identity information?
Q38: Do the healthcare devices ensure the privacy of data related to patients?
Q39: What is the level of privacy protection furnished by existing IoHT applications?
Q40: Rate the privacy protection features in overall authentication processes
Feature: Forward security
Q41: What is the role of forward security in exposing the session key?
Q42: How previous sessions are protected from future threats?
Q43: How does this feature provide resilience against different attacks?

**Figure 3:**
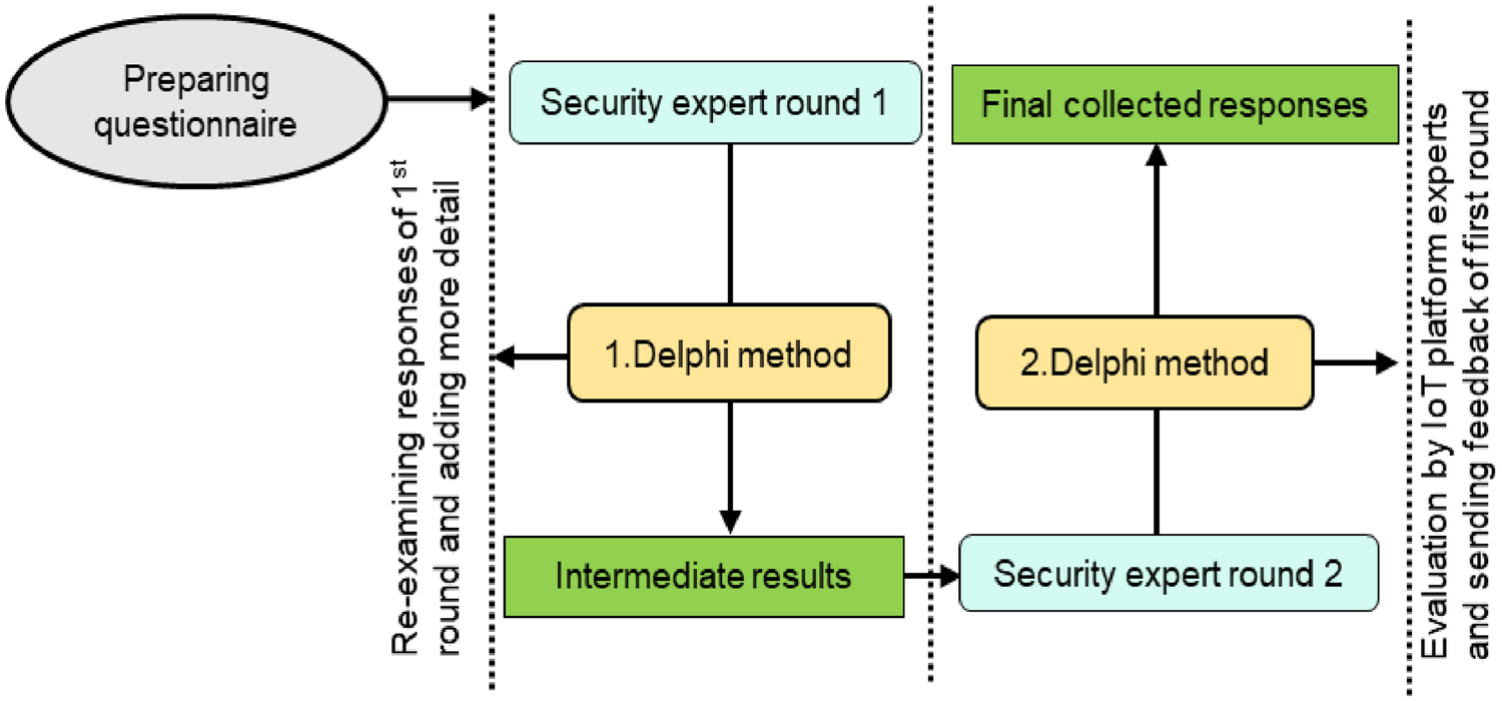
Application of the Delphi method for data collection.

Security experts rated the importance of features in authentication schemes based on their expert opinions. The responses of the experts about the authentication features were obtained by using a well-known scale Saaty’s scale. According to experts and literature studies, the most important features of authentication are mutual authentication, availability, integrity, privacy protection, key agreement, password change, confidentiality, forward security, and scalability. The method of data collection is based on the application of the Delphi method. This process is completed in two different rounds. The detail of using the Delphi method is given in Fig. 3. The security evaluation criteria are created according to the collected features. These security requirements are essential for healthcare-related data^38^.

The selected features of the proposed evaluation framework are discussed below.

- Mutual authentication (C_1_)

Mutual authentication involves the procedure of verifying the identities of two parties or entities involved in the secure authentication. Robust mutual authentication is vital to thwart man-in-the-middle attacks in a medical environment.

- Privacy Protection(C_2_)

It is important to keep secret sensitive data about patients or medical records from outside the world like hackers, companies, third parties, or other groups.

- Key agreement (C_3_)

It is an implicit authentication process where two or more two communication parties based on using similar keys achieve secure communication.

- Password change(C_4_)

The client needs to change their old credential in the scenario when a security breach is encountered in the network.

- Integrity (C_5_)

Integrity means data should not be altered by unlawful modifications. The patients’ data need to be in correct and complete form in the healthcare environment^39^.

- Availability(C_6_)

It specifies that all the important services and information need to be available to authentic users in a timely and effective way. Availability ensures that when data or devices are to be accessed, it will not malfunction or access will not be denied ^40^.

- Confidentiality (C_7_)

Confidentiality ensures that an authorized entity or procedure has access to the information resources and network^41^. It is mandatory to secure the sensitive data related to the patients from outside access during the procedure of transmitting data to the processing system via communication link like Wi-Fi or cellular network

- Forward security(C_8_)

Forward security is the most important security attribute for key exchange and authentication schemes. Forward security provides a strong defense against the file-injection type attacks. Modern authentication protocols or schemes are based on forward security ^42,43^.

- Scalability(C_9_)

The scalability of authentication is also an important feature and it is dependent on the key-block size as the key-block size increases then scalability is also increased exponentially^44^. In the latest introduced authentication protocol scalability and efficiency are the most prominent features ^45,46^.

The selected features are collected according to the frequency of occurrence and commonality in the literature. following authentication features from the literature sources are collected as shown in Table 3.

**Table 3:** Criteria features and citations.

Features	Citation
Mutual authentication	^47–59^
Privacy protection	^17,47,55,56,60,61^
Key agreement	^49,57–59,61,62^
Password change	^54,57,58,63^
Integrity	^17,55,57,58,61,64–68^
Confidentiality	^17,48,50,52,55,62,64–69^
Forward Security	^59,62,63,67^
Scalability	^57,62,68,70^
Availability	^17,56,59,62,64,65^

**Figure 4:**
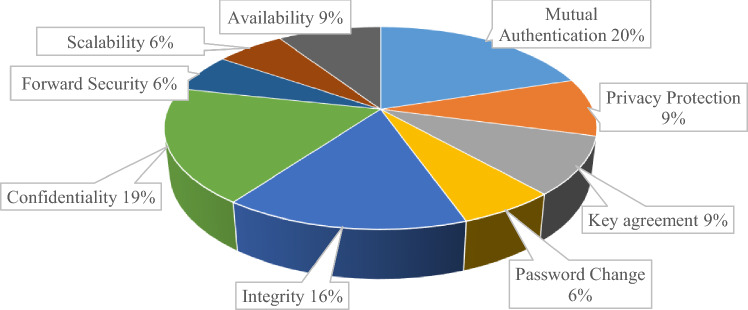
Criteria features in the existing literature.

The detail of each feature based on the literature occurrence is given in Fig. 4.

- Variable selection

This is the initial and very crucial step, where the major focus is to select the most relevant and important variable regarding the research survey questions. The prevalent and unimportant variables were discarded by adopting the features selection method.

- Data cleaning

The data cleaning is very important before inputting the data for analysis. The outliners in the collected data are removed by following the well-known approaches such Winsorization, imputation methods and sensitivity analysis.

- Data formatting

During the data formatting step, the collected data were divided into numerical and categorical ways to perform the data analysis and visualization.

- Data coding

As the survey has been carried out by presenting the open-ended questions to the expert panel. The collected responses were given numerical codes by following the manual procedure of coding.

### Graph theory and matrix approach

The Graph Theory & Matrix (GTM) approach follows a mathematical operation for analysis, evaluation, and decision-making^7^. GTM models variable relationships using graph theory, with nodes representing variables and edges representing interactions. This graphical depiction helps with the visual study and interpretation of complicated systems. By comparing the GTM approach with similar approaches like Bayesian networks and Structural Equation Modeling (SEM), GTM has several advantages. In contrast to these methodologies, GTM emphasizes visual depiction and intuitive exploration of system dynamics using graph-theoretic principles. It provides a novel perspective that complements established quantitative methods, making it ideal for modeling complex systems with interrelated components. Bayesian networks model interactions between variables, but they use probabilistic graphical models to depict dependencies and infer causal linkages from observable data. SEM is a method of analyzing the links between observable and latent variables using a system of equations. This allows complex theoretical models to be tested. The GTM approach consists of the following phases after finalizing the alternatives and security features^71,72^.

*Phase-1*: This method represents the data items in a digraph fashion which is very beneficial for modeling and analysing the various types of systems in the area of science and technology. A digraph is the type of graph denoted by the directed edges which are connecting the nodes. A digraph involves different nodes and edges.

Definition: Digraph is an ordered pair of sets “G”. This graph can be mathematically written by using Eq. (1):

1 $$G=(V,E)$$

In Eq. (1), the set of vertices/nodes and edges/arcs are denoted by “V” and “E”, respectively. The set of nodes and edges are given below mathematically.

2 $${V=\{v}_{i} \mathrm{where \,i}=\mathrm{1,2},\mathrm{3,4}\dots .\mathrm{m \,}\& \,{E=\{E}_{ij}\}$$

*Phase 2*: In the second step, the GTM approach represents the performance of attributes digraph into one-to-one matrix form. This matrix is called the performance attributes matrix (PAM), it is very helpful during the analysis of digraph expeditiously to derive the system functions. It is a M × N matrix that considers all of the attributes and their relative importance. The PAM is given by Eq. (3).

3 $$PAM=\left[\begin{array}{ccccccccc}{D}_{1}& {d}_{12}& {d}_{13}& {d}_{14}& {d}_{15}& {d}_{16}& {d}_{17}& {d}_{17}& {d}_{18}\\ {d}_{21}& {D}_{2}& {d}_{23}& {d}_{24}& {d}_{25}& {d}_{26}& {d}_{27}& {d}_{28}& {d}_{29}\\ {d}_{31}& {d}_{32}& {D}_{3}& {d}_{34}& {d}_{35}& {d}_{36}& {d}_{37}& {d}_{38}& {d}_{39}\\ {d}_{41}& {d}_{42}& {d}_{43}& {D}_{4}& {d}_{45}& {d}_{46}& {d}_{47}& {d}_{48}& {d}_{49}\\ {d}_{51}& {d}_{52}& {d}_{53}& {d}_{54}& {D}_{5}& {d}_{56}& {d}_{57}& {d}_{58}& {d}_{59}\\ {d}_{61}& {d}_{62}& {d}_{63}& {d}_{64}& {d}_{65}& {D}_{6}& {d}_{67}& {d}_{68}& {d}_{69}\\ {d}_{71}& {d}_{72}& {d}_{73}& {d}_{74}& {d}_{75}& {d}_{76}& {D}_{7}& {d}_{78}& {d}_{79}\\ {d}_{81}& {d}_{82}& {d}_{83}& {d}_{84}& {d}_{85}& {d}_{86}& {d}_{87}& {D}_{8}& {d}_{89}\\ {d}_{91}& {d}_{92}& {d}_{93}& {d}_{94}& {d}_{95}& {d}_{96}& {d}_{97}& {d}_{98}& {D}_{9}\end{array}\right]$$

*Phase 3*: In this step, the permanent matrix is a standard matrix function that has wider applications in combinatorial mathematics. The permanent function is calculated in a similar procedure as the determinant of a matrix is obtained but has all positive signs. It is very helpful as it produces better results, and no information is lost due to the involvement of positive signs of the permutations. The permanent of the matrix (P_m_) is given in Eq. (4).

4 $$\begin{aligned}{{\text{P}}}_{{\text{m}}}& \text{=} \prod_{{\text{i}}=1}^{{\text{M}}}{{\text{D}}}_{{\text{i}}} +\sum_{{\text{i}}=1}^{{\text{M}}-1}\sum_{{\text{j}}={\text{i}}+1}^{{\text{M}}}\dots \sum_{{\text{M}}={\text{T}}+1}^{{\text{M}}} \left({{\text{d}}}_{{\text{ij}}}{{\text{d}}}_{{\text{ji}}}\right){{\text{D}}}_{{\text{k}}}{{\text{D}}}_{{\text{l}}}{{\text{D}}}_{{\text{m}}}{{\text{D}}}_{{\text{n}}}{{\text{D}}}_{{\text{o}}}\dots {{\text{D}}}_{{\text{t}}}{{\text{D}}}_{{\text{m}}} \\ & \quad + \sum_{{\text{i}}=1}^{{\text{M}}-2}\sum_{{\text{j}}={\text{i}}+1}^{{\text{M}}-1}\sum_{{\text{k}}={\text{j}}+1}^{{\text{M}}-1} + \sum_{{\text{M}}={\text{t}}+1}^{{\text{m}}}\begin{array}{c}\left({{\text{d}}}_{{\text{ij}}}{{\text{d}}}_{{\text{jk}}}{{\text{d}}}_{{\text{ki}}}+{{\text{d}}}_{{\text{ik}}}{{\text{d}}}_{{\text{kj}}}{{\text{d}}}_{{\text{ji}}}\right){{\text{D}}}_{{\text{k}}}{{\text{D}}}_{{\text{l}}}{{\text{D}}}_{{\text{m}}}{{\text{D}}}_{{\text{n}}}{{\text{D}}}_{{\text{o}}}\dots {{\text{D}}}_{{\text{t}}}{{\text{D}}}_{{\text{m}}}\\ \end{array} \\ & \quad + \left[\begin{array}{c} \\ \sum_{{\text{i}}=1}^{{\text{M}}-3} \sum_{{\text{j}}={\text{i}}+1}^{{\text{M}}}\sum_{{\text{k}}={\text{I}}+1}^{{\text{M}}-1}\sum_{{\text{l}}={\text{i}}+2}^{{\text{M}}-1} + \sum_{{\text{M}}={\text{t}}+1}^{{\text{m}}}\left({{\text{d}}}_{{\text{ij}}}{{\text{d}}}_{{\text{ji}}}\right)\left({{\text{d}}}_{{\text{kl}}}{{\text{d}}}_{{\text{lk}}}\right){{\text{D}}}_{{\text{m}}}{{\text{D}}}_{{\text{n}}}{{\text{D}}}_{{\text{O}}}.{{\text{D}}}_{{\text{t}}}{{\text{D}}}_{{\text{m}}} + \\ \sum_{{\text{i}}=1}^{{\text{M}}-3} \sum_{{\text{j}}={\text{i}}+1}^{{\text{M}}-1}+\sum_{{\text{M}}={\text{t}}+1}^{{\text{m}}}\left({{\text{d}}}_{{\text{ij}}}{{\text{d}}}_{{\text{jk}}}{{\text{d}}}_{{\text{kl}}}{{\text{d}}}_{{\text{li}}}+{{\text{d}}}_{{\text{il}}}{{\text{d}}}_{{\text{lk}}}{{\text{d}}}_{{\text{kj}}}{{\text{d}}}_{{\text{ji}}}\right){{\text{D}}}_{{\text{m}}}{{\text{D}}}_{{\text{n}}}{{\text{D}}}_{{\text{O}}}{{\text{D}}}_{{\text{t}}}{{\text{D}}}_{{\text{m}}}\end{array}\right]\\ & \quad+\left[\sum_{{\text{i}}=1}^{{\text{M}}-2} \sum_{{\text{j}}={\text{i}}+1}^{{\text{M}}-1}\sum_{{\text{k}}={\text{j}}+1}^{{\text{M}}}\sum_{{\text{l}}=1}^{{\text{M}}-1} \sum_{{\text{m}}={\text{l}}+1}^{{\text{M}}}\dots ..+\sum_{{\text{M}}={\text{t}}+1}^{{\text{m}}}\left({{\text{d}}}_{{\text{ij}}}{{\text{d}}}_{{\text{jk}}}{{\text{d}}}_{{\text{kl}}}{{\text{d}}}_{{\text{li}}}+{{\text{d}}}_{{\text{il}}}{{\text{d}}}_{{\text{lk}}}{{\text{d}}}_{{\text{kj}}}{{\text{d}}}_{{\text{ji}}}\right)\left({{\text{d}}}_{{\text{lm}}}{{\text{d}}}_{{\text{ml}}}\right){{\text{D}}}_{{\text{m}}}{{\text{D}}}_{{\text{n}}}{{\text{D}}}_{{\text{O}}}\dots ...{{\text{D}}}_{{\text{t}}}{{\text{D}}}_{{\text{m}}} \right]\\ & \quad +\left[\sum_{{\text{i}}=1}^{{\text{M}}-4} \sum_{{\text{j}}={\text{i}}+1}^{{\text{M}}-1}\sum_{{\text{k}}={\text{i}}+1}^{{\text{M}}}\sum_{{\text{l}}={\text{i}}+1}^{{\text{M}}} \sum_{{\text{m}}={\text{j}}+1}^{{\text{M}}} \dots .+ \sum_{{\text{M}}={\text{t}}+1}^{{\text{M}}}\left({{\text{d}}}_{{\text{ij}}}{{\text{d}}}_{{\text{jk}}}{{\text{d}}}_{{\text{kl}}}{{\text{d}}}_{{\text{lm}}}{{\text{d}}}_{{\text{mi}}}+{{\text{d}}}_{{\text{im}}}{{\text{d}}}_{{\text{ml}}}{{\text{d}}}_{{\text{lk}}}{{\text{d}}}_{{\text{kj}}}{{\text{d}}}_{{\text{ji}}}\right){{\text{D}}}_{{\text{n}}}{{\text{D}}}_{{\text{O}}}\dots \dots .{{\text{D}}}_{{\text{t}}}{{\text{D}}}_{{\text{m}}} \right]\\ & \quad +\left[\sum_{{\text{i}}=1}^{{\text{M}}-3} \sum_{{\text{j}}={\text{i}}+1}^{{\text{M}}-1}\sum_{{\text{k}}={\text{i}}+1}^{{\text{M}}}\sum_{{\text{l}}={\text{j}}+1}^{{\text{M}}} \sum_{{\text{m}}=1}^{{\text{M}}-1}\sum_{{\text{n}}={\text{m}}+1}^{{\text{M}}}. .... \sum_{{\text{M}}={\text{t}}+1}^{{\text{M}}}\left({{\text{d}}}_{{\text{ij}}}{{\text{d}}}_{{\text{jk}}}{{\text{d}}}_{{\text{kl}}}{{\text{d}}}_{{\text{li}}}+{{\text{d}}}_{{\text{il}}}{{\text{d}}}_{{\text{lk}}}{{\text{d}}}_{{\text{kj}}}{{\text{d}}}_{{\text{ji}}}\right)\left({{\text{d}}}_{{\text{mn}}}{{\text{d}}}_{{\text{nm}}}\right){{\text{D}}}_{{\text{O}}}\dots ..{{\text{D}}}_{{\text{t}}}{{\text{D}}}_{{\text{m}}} \right]\\ & \quad +\left[\sum_{{\text{i}}=1}^{{\text{M}}-5} \sum_{{\text{j}}={\text{i}}+1}^{{\text{M}}-1}\sum_{{\text{k}}={\text{j}}+1}^{{\text{M}}}\sum_{{\text{l}}=1}^{{\text{M}}} \dots \sum_{{\text{M}}={\text{t}}+1}^{{\text{M}}}\left({{\text{d}}}_{{\text{ij}}}{{\text{d}}}_{{\text{jk}}}{{\text{d}}}_{{\text{ki}}}+{{\text{d}}}_{{\text{ik}}}{{\text{d}}}_{{\text{kj}}}{{\text{d}}}_{{\text{kj}}}{{\text{d}}}_{{\text{ji}}}\right)\left({{{\text{d}}}_{{\text{lm}}}{\text{d}}}_{{\text{mn}}}{{\text{d}}}_{{\text{nl}}}+{{{\text{d}}}_{{\text{ln}}}{\text{d}}}_{{\text{nm}}}{{\text{d}}}_{{\text{ml}}}\right){{\text{D}}}_{{\text{O}}}\dots \dots .{{\text{D}}}_{{\text{t}}}{{\text{D}}}_{{\text{m}}}\right. \\ & \quad \left.+\sum_{{\text{i}}=1}^{{\text{M}}-5}\sum_{{\text{j}}={\text{i}}+1}^{{\text{M}}} \sum_{{\text{k}}={\text{i}}+1}^{{\text{M}}-3}\sum_{{\text{l}}={\text{i}}+2}^{{\text{M}}} \sum_{{\text{m}}={\text{k}}+1}^{{\text{M}}-1}.. \sum_{{\text{m}}={\text{t}}+1}^{{\text{M}}} \left({{\text{d}}}_{{\text{ij}}}{{\text{d}}}_{{\text{ji}}}\right)\left({{\text{d}}}_{{\text{kl}}}{{\text{d}}}_{{\text{lk}}}\right){({\text{d}}}_{{\text{m}}}{{\text{d}}}_{{\text{n}}}){{\text{D}}}_{{\text{O}}} {{\text{D}}}_{{\text{t}}}{{\text{D}}}_{{\text{m}}}+\sum_{{\text{i}}=1}^{{\text{M}}-5}\sum_{{\text{j}}={\text{i}}+1}^{{\text{M}}-1} \sum_{{\text{k}}={\text{i}}+1}^{{\text{M}}}\sum_{{\text{l}}={\text{i}}+1}^{{\text{M}}} \sum_{{\text{m}}={\text{i}}+1}^{{\text{M}}}\sum_{{\text{n}}={\text{j}}+1}^{{\text{M}}}\right.\\ & \quad \left.\dots ..\sum_{{\text{M}}={\text{t}}+1}^{{\text{M}}} ({{\text{d}}}_{{\text{ij}}}{{\text{d}}}_{{\text{jk}}}{{\text{d}}}_{{\text{kl}}}{{\text{d}}}_{{\text{lm}}}{{\text{d}}}_{{\text{mn}}}{{\text{d}}}_{{\text{ni}}}+{{\text{d}}}_{{\text{in}}}{{\text{d}}}_{{\text{nm}}}{{\text{d}}}_{{\text{ml}}}{{\text{d}}}_{{\text{lk}}}{{\text{d}}}_{{\text{kj}}}{{\text{d}}}_{{\text{ji}}}){{\text{D}}}_{{\text{O}}}{{\text{D}}}_{{\text{t}}}{{\text{D}}}_{{\text{m}}}\right]\end{aligned}$$

The permanent performance index among the attributes is obtained by finding the relative importance. The relative importance (a_ji_) is given based on the scale ranges between 0 and 1. The value of relative importance is calculated by Eq. (5).

5 $${a}_{ji}=\frac{1}{aij} \,or \,1-{a}_{ij}$$

The GTM approach consists of the following steps for the assessment and selection of a secure authentication method or solution.

#### Step-1: Identifying features and alternatives

The main purpose of using the GTM approach is to evaluate the authentication solutions for IoT devices based on the identified features in the medical care system. For the evaluation, this study assumes ten (10) authentication protocols for IoT devices concerning identified authentication features. As mentioned earlier, nine (9) authentication features i.e. mutual authentication, key agreement, password change, integrity, privacy protection, confidentiality, forward security, scalability, and availability are selected. In this proposed authentication evaluation framework, the selected features are written concerning ten (10) selected authentication alternatives due to the number of security experts involved.

#### Step 2: Graph representation of authentication features

**Figure 5:**
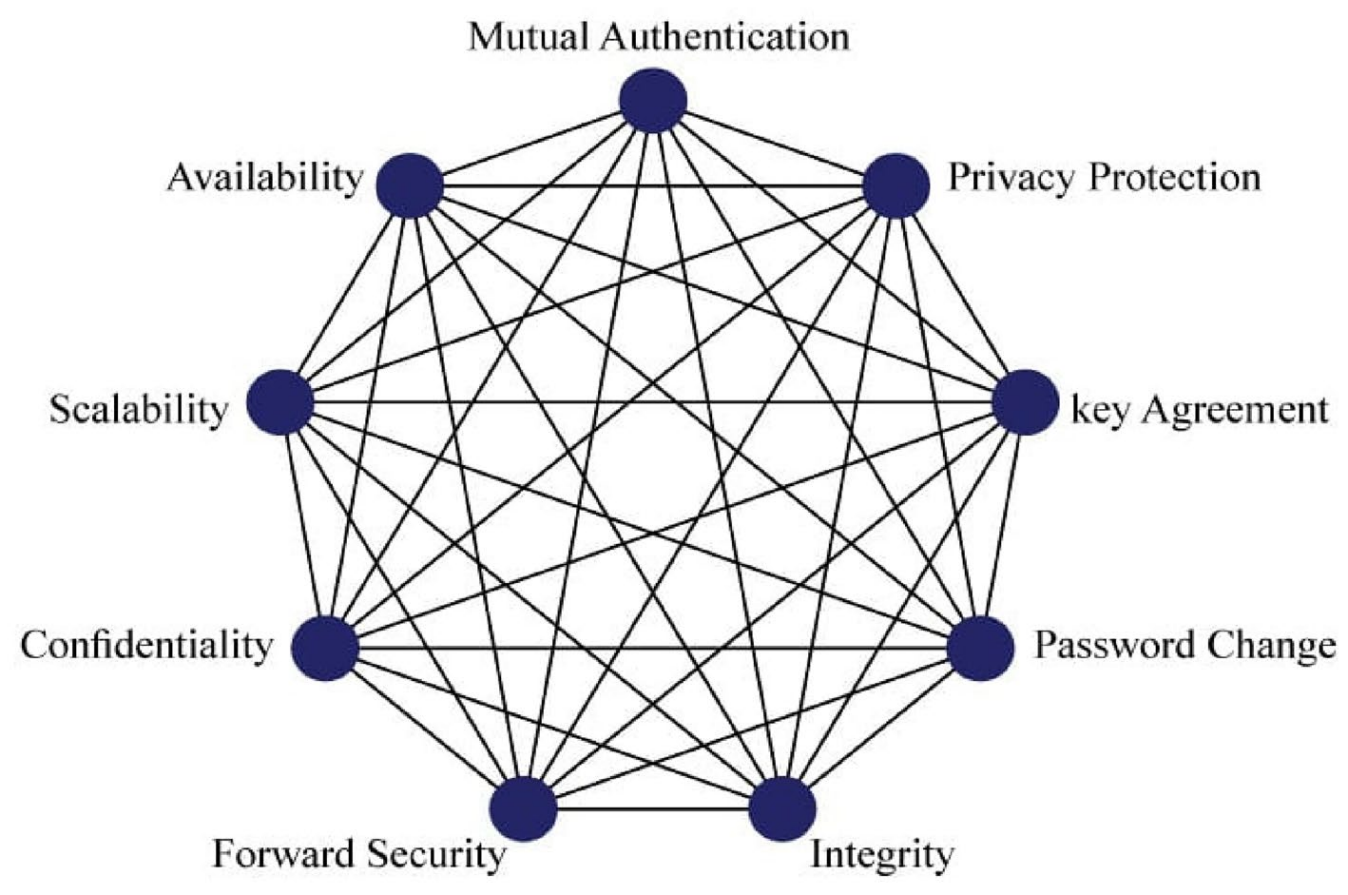
Features digraph representation.

In this step, security features or attributes are represented in the form of digraphs. All attributes are written in nodes and edges are shown the interdependencies among the security features. The digraph authentication features are shown in Fig. 5.

#### Step 3: Building decision matrix and Permanent function

The decision matrix is built by performing a well-organized case study and interviewing the IoT security expert. Data is collected based on the importance of these features for IoT devices, which features are important and how they affect the authentication mechanism or scheme, which features to include, and which ones to less important under different circumstances in the healthcare environment. The expert described these features in linguistic terms. Saaty’s scale is used for converting linguistic terms into integer values. Data related to different authentication features is presented in the form of an input matrix by experts. The data collected from experts is arranged in the form of authentication alternatives which are denoted by (A_1_, A_2_, A_3,_ A_4,_ A_5,_ A_6,_ A_7,_ A_8,_ A_9,_ and A_10_). The security features are coded for simplicity such as mutual authentication, privacy protection, key agreement, password change, integrity, confidentiality, forward security, scalability, and availability are coded as *C*_*1*_*, C*_*2*_*, C*_*3*_*, C*_*4*_*, C*_*5*_*, C*_*6*_*, C*_*7*_*, C*_*8,*_ and* C*_*9*_ respectively. Data is provided in the decision matrix by the expert panel against the features given.

$${D}_{m}=\left[\begin{array}{cccccccccc} \, & {\text{C}}_{1}& {\text{C}}_{2}& {\text{C}}_{3}& {\text{C}}_{4}& {\text{C}}_{5}& {\text{C}}_{6}& {\text{C}}_{7}& {\text{C}}_{8}& {\text{C}}_{9}\\ {\text{A}}_{1}& {7}& {7}& {6}& {8}& {6}& {8}& {5}& {6}& {9}\\ {\text{A}}_{2}& {7}& {6}& {7}& {7}& {7}& {6}& {5}& {5}& {9}\\ {\text{A}}_{3}& {8}& {6}& {6}& {8}& {8}& {7}& {4}& {6}& {7}\\ {\text{A}}_{4}& {7}& {7}& {8}& {6}& {9}& {7}& {6}& {5}& {6}\\ {\text{A}}_{5}& {5}& {6}& {6}& {7}& {8}& {6}& {7}& {5}& {8}\\ {\text{A}}_{6}& {6}& {7}& {6}& {8}& {6}& {8}& {5}& {8}& {9}\\ {\text{A}}_{7}& {6}& {7}& {6}& {8}& {6}& {8}& {5}& {8}& {9}\\ {\text{A}}_{8}& {6}& {8}& {7}& {4}& {8}& {6}& {8}& {7}& {6}\\ {\text{A}}_{9}& {7}& {7}& {5}& {8}& {9}& {9}& {6}& {4}& {8}\\ {\text{A}}_{10}& {7}& {6}& {7}& {4}& {6}& {7}& {5}& {4}& {8}\end{array}\right]$$

A normalized decision Matrix (N_dm_) is obtained to remove the element of biases as data in this matrix come from the different expert’s opinions. This matrix is built with the help of an expert panel as shown below.

$${N}_{dm}=\left[\begin{array}{cccccccccc} \, & {\text{C}}_{1}& {\text{C}}_{2}& {\text{C}}_{3}& {\text{C}}_{4}& {\text{C}}_{5}& {\text{C}}_{6}& {\text{C}}_{7}& {\text{C}}_{8}& {\text{C}}_{9}\\ {\text{A}}_{1}& \text{0.88}& \text{0.88}& \text{0.75}& {1}& \text{0.67}& \text{0.89}& \text{0.63}& \text{0.75}& {1}\\ {\text{A}}_{2}& \text{0.88}& \text{0.75}& \text{0.88}& \text{0.88}& \text{0.78}& \text{0.67}& \text{0.63}& \text{0.63}& {1}\\ {\text{A}}_{3}& {1}& \text{0.75}& \text{0.75}& {1}& \text{0.89}& \text{0.78}& \text{0.5}& \text{0.75}& \text{0.78}\\ {\text{A}}_{4}& \text{0.88}& \text{0.88}& {1}& \text{0.75}& {1}& \text{0.78}& \text{0.75}& \text{0.63}& \text{0.68}\\ {\text{A}}_{5}& \text{0.63}& \text{0.75}& \text{0.75}& \text{0.88}& \text{0.89}& \text{0.67}& \text{0.88}& \text{0.63}& \text{0.89}\\ {\text{A}}_{6}& \text{0.75}& \text{0.88}& \text{0.75}& {1}& \text{0.67}& \text{0.89}& \text{0.63}& {1}& {1}\\ {\text{A}}_{7}& \text{0.63}& \text{0.88}& \text{0.63}& {1}& \text{0.78}& {1}& \text{0.75}& \text{0.63}& \text{0.89}\\ {\text{A}}_{8}& \text{0.75}& {1}& \text{0.88}& \text{0.5}& \text{0.89}& \text{0.67}& {1}& \text{0.88}& \text{0.67}\\ {\text{A}}_{9}& \text{0.88}& \text{0.88}& \text{0.63}& {1}& {1}& {1}& \text{0.75}& \text{0.5}& \text{0.89}\\ {\text{A}}_{10}& \text{0.88}& \text{0.75}& \text{0.88}& \text{0.5}& \text{0.67}& \text{0.78}& \text{0.63}& \text{0.50}& \text{0.89}\end{array}\right]$$

To obtain the permanent matrix, the values of the normalized decision matrix are determined. The permanent functions calculated for every alternative are listed in Table 4. Based on the value of permanent functions the alternatives ranking is performed.

**Table 4:** Ranking alternatives.

Alternatives	Permanent matrix	Prioritization
A_1_	1007.9	3
A_2_	928.1	8
A_3_	948.457	6
A_4_	978.545	4
A_5_	903.483	9
A_6_	1044.55	1
A_7_	947.476	7
A_8_	966.776	5
A_9_	1014.68	2
A_10_	807.148	10

From the results of Table 4, it is evident that the A_6_ alternative has achieved the higher values among the list of selected authentication solution alternatives. So, it is considered the best security solution alternative for IoT devices in the IoHT environment in terms of defined feature-based criteria. Now, it is important to know about the input values provided against the higher-ranked alternative. From this, it is concluded that features are affecting the assessment and ranking process of selection and ranking authentication schemes in the healthcare environment.

## Results and discussion

**Figure 6:**
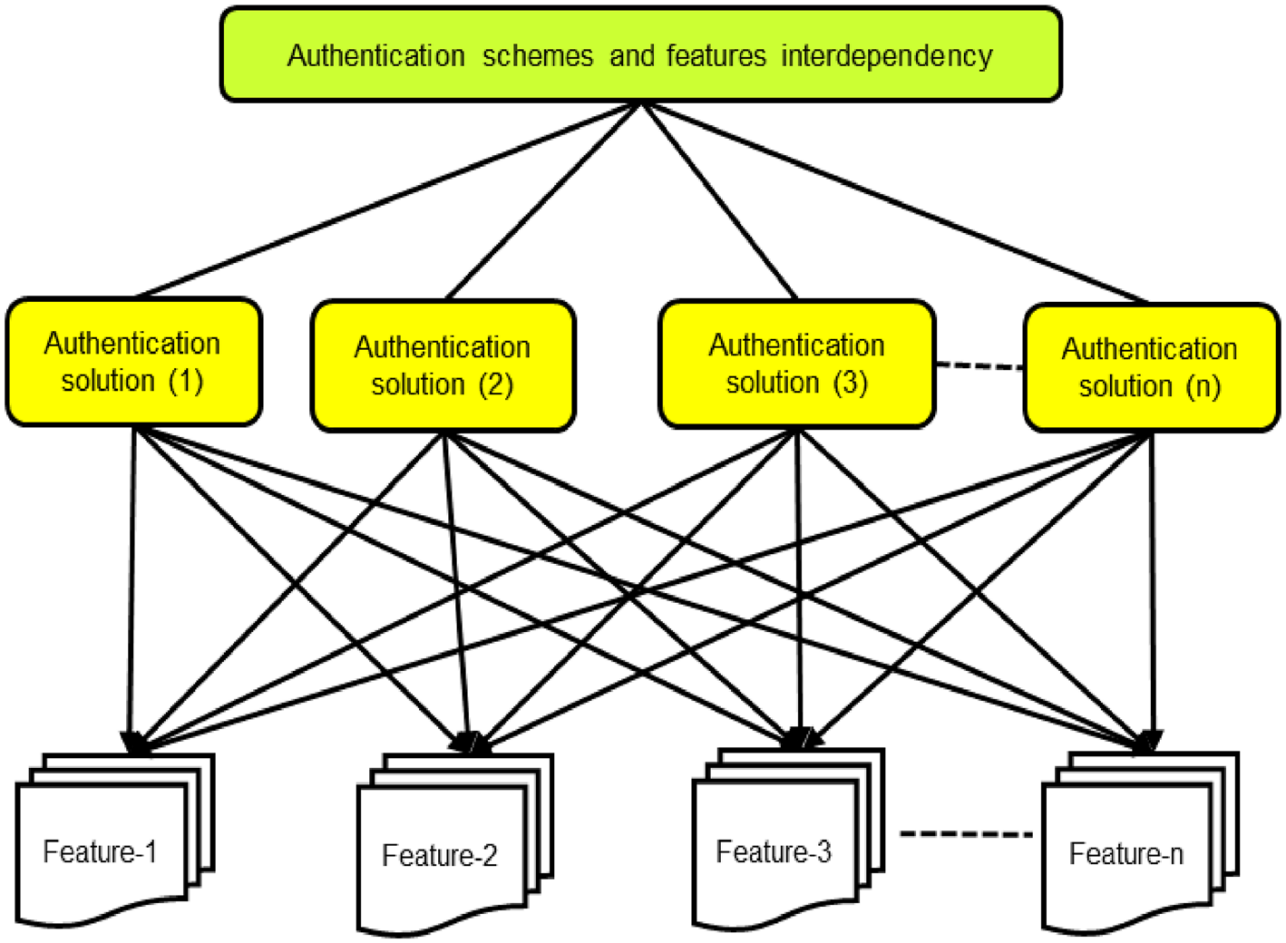
Authentication schemes and features inter-dependencies.

The recommended framework is validated by using hybrid MCDM techniques such as AHP and TOPSIS. This method is presented by Hwang &Yoon^73^ which is making decisions based on using the ideal solution, for instance, if a particular alternative is closer to the positive ideal solution then it will be reckoned as the best and most appropriate solution. It follows a simple computation procedure supported by reliability and well-establishment characteristics^73^. According to the TOPSIS method, the selected choice should have the minimum distance from the positive ideal solution and the maximum distance from the negative ideal solution. TOPSIS and AHP are more idealistic in situations, where the features and alternatives are interdependent. In the proposed model, the hierarchical relationships between alternatives and features are given in Fig. 6.

The detail of all symbol parameters is given in Table 5.

**Table 5:** Symbols Description.

Symbol	Description
Cn	Number of criteria
An	Number of alternatives
D_M_	Decision matrix
D_NM_	Normalized decision matrix
W_NDM_	Weighted Normalized decision matrix
A^+^	Ideal positive solution
A^-^	Negative ideal solution
S^+^	Ideal separation measure
S^-^	Non-ideal separation measure
C_i_	Consistency index or relative closeness

TOPSIS method adopts the following procedure as shown in Table 6^73,74^.

**Table 6:**
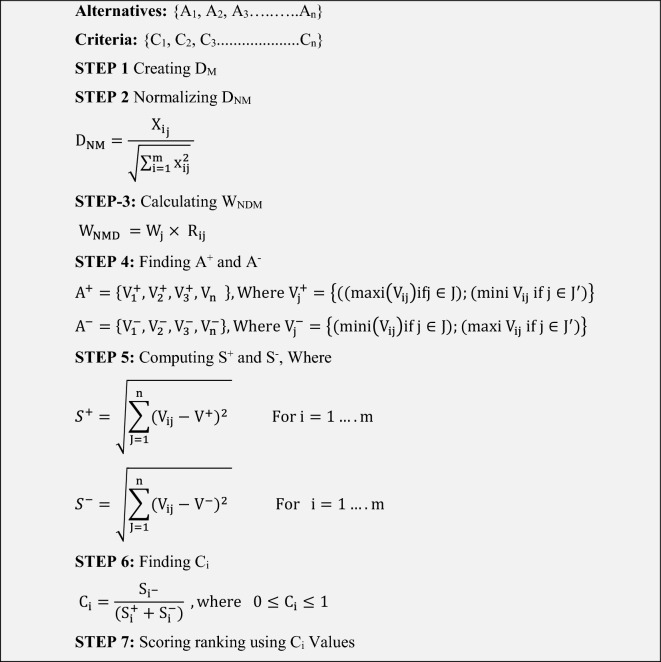
Algorithm steps.

The TOSIS method has been applied to check the validity of the proposed IoHT assessment authentication framework based on the authentication features. This method validates the results obtained from the GTM approach. The previously collected data has been provided as input in the form of a decision matrix for the TOPSIS method. The decision matrix is composed of the values assigned by the expert panel against the features. The weights are assigned to the authentication feature by the expert panel in qualitative form, and then they are converted to numeric form by using Saaty’s scale. The values are assigned based on Saaty’s scale, starting from 0 to 10, for each alternative against the security features by the experts. The details of the values assigned by a group of ten (10) expert panels to the alternatives and features are depicted in the matrix (Dm), given as.

$${\text{D}}_{\text{m}}= \left[\begin{array}{cccccccccc} \, & {\text{C}}_{1}& {\text{C}}_{2}& {\text{C}}_{3}& {\text{C}}_{4}& {\text{C}}_{5}& {\text{C}}_{6}& {\text{C}}_{7}& {\text{C}}_{8}& {\text{C}}_{9}\\ {\text{A}}_{1}& {7}& {7}& {6}& {8}& {6}& {8}& {5}& {6}& {9}\\ {\text{A}}_{2}& {7}& {6}& {7}& {7}& {7}& {6}& {5}& {5}& {9}\\ {\text{A}}_{3}& {8}& {6}& {6}& {8}& {8}& {7}& {4}& {6}& {7}\\ {\text{A}}_{4}& {7}& {7}& {8}& {6}& {9}& {7}& {6}& {5}& {6}\\ {\text{A}}_{5}& {5}& {6}& {6}& {7}& {8}& {6}& {7}& {5}& {8}\\ {\text{A}}_{6}& {6}& {7}& {6}& {8}& {6}& {8}& {5}& {8}& {9}\\ {\text{A}}_{7}& {6}& {7}& {6}& {8}& {6}& {8}& {5}& {8}& {9}\\ {\text{A}}_{8}& {6}& {8}& {7}& {4}& {8}& {6}& {8}& {7}& {6}\\ {\text{A}}_{9}& {7}& {7}& {5}& {8}& {9}& {9}& {6}& {4}& {8}\\ {\text{A}}_{10}& {7}& {6}& {7}& {4}& {6}& {7}& {5}& {4}& {8}\end{array}\right]$$

After creating the decision matrix which represents criteria and features. The next step is to apply the algorithm as given in Table 5. The weights of the criteria features are the most important step. To avoid the element of subjectivity and biases, AHP is applied which is a well-known technique. Finally, with the help of the TOPSIS approach, the Relative closeness values are determined which is very effective in prioritizing the alternatives. The results of the application of the TOPSIS approach are given in Table 7.

**Table 7:** Ideal separation measures and relative closeness.

Alternatives	S^+^	S^-^	${{\text{S}}}^{+}+{{\text{S}}}^{-}$	Relative closeness (R.C)
A_1_	0.026	0.037	0.064	0.585
A_2_	0.031	0.029	0.060	0.479
A_3_	0.031	0.035	0.066	0.526
A_4_	0.027	0.027	0.054	0.500
A_5_	0.026	0.033	0.059	0.556
A_6_	0.024	0.041	0.065	0.628
A_7_	0.024	0.041	0.065	0.627
A_8_	0.024	0.038	0.062	0.612
A_9_	0.037	0.032	0.069	0.459
A_10_	0.026	0.040	0.066	0.606

Finally, the ranking or prioritization of alternatives is given in Table 8. In Table 8, the A6 alternative has a higher value and is first in rank among all other alternatives based on authentication security features, so it can be described as the most reliable and secure IoT solution for an IoT-based healthcare environment.

**Table 8:** Ranking alternatives.

Alt	A_1_	A_2_	A_3_	A_4_	A_5_	A_6_	A_7_	A_8_	A_9_	A_10_
R.C	0.585	0.479	0.526	0.5	0.556	0.628	0.627	0.612	0.459	0.606
Ranking	5	9	7	8	6	1	2	3	10	4

**Figure 7:**
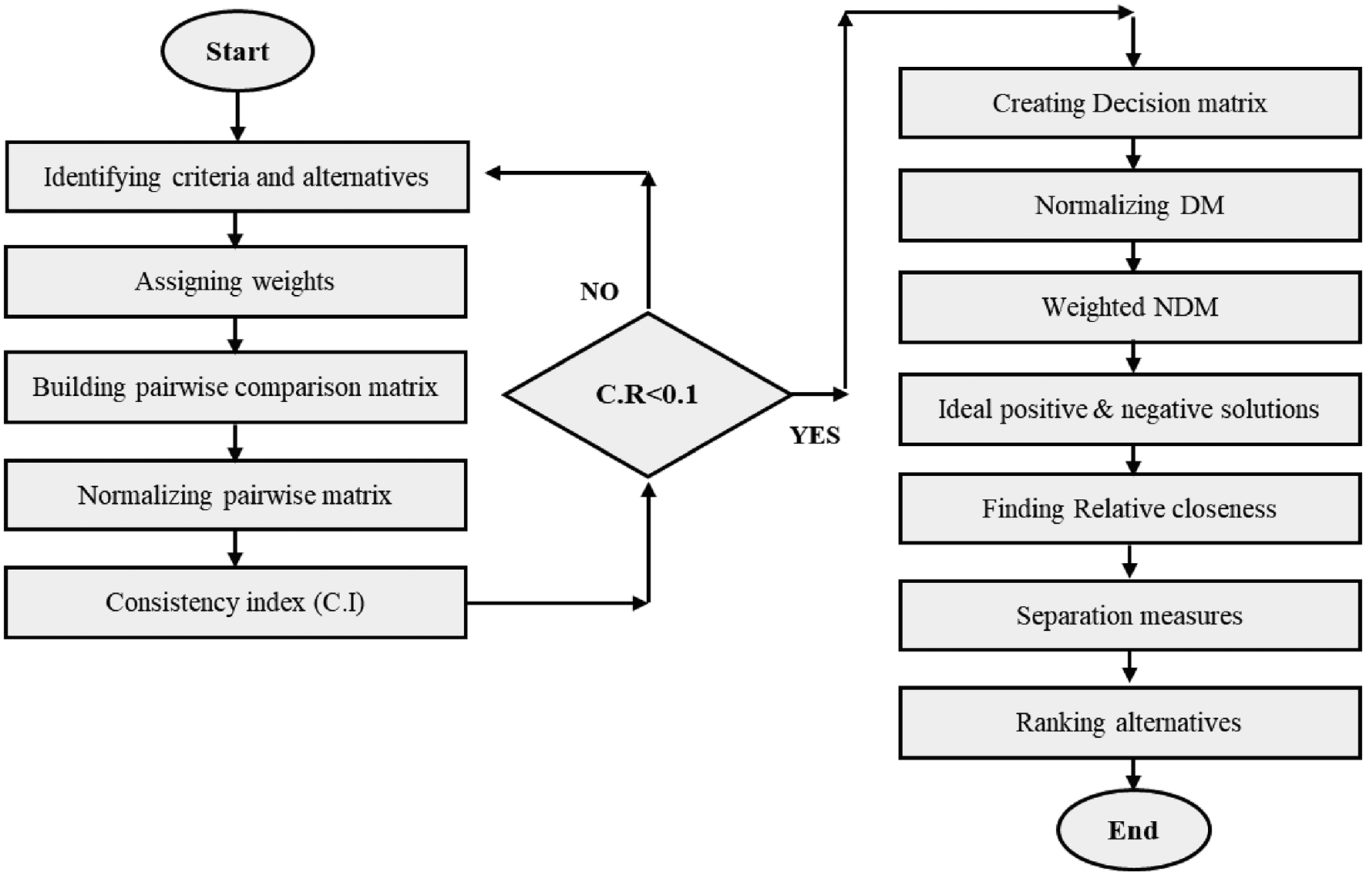
Flowchart of validation approach.

The flowchart diagram of the integrated approach AHP-TOPSIS to validate the proposed evaluation framework is given in Fig. 7.

The recommended framework is validated by using an integrated approach of AHP-TOPSIS techniques. The major purpose is to check the accuracy and consistency of results obtained from the previously applied method (GTM). Among the assumed alternatives, A_6_ has the higher value among the alternatives. Hence, the assessment and ranking done by the GTM approach is validated and results are precise and accurate based on the validation of TOPSIS. The results comparison of both GTM and TOPSIS approaches are graphically given in Table 9.

**Table 9:** Comparison of proposed work with other techniques.

TOPSIS method			Proposed work (GTM)	
Alt(s)	Ranking score(S_i_)	Ranking	Permanent matrix	Ranking
A_1_	0.585	5	1007.9	3
A_2_	0.479	9	928.1	8
A_3_	0.526	7	948.457	6
A_4_	0.500	8	978.545	4
A_5_	0.556	6	903.483	9
A6	0.628	1	1044.55	1
A7	0.627	2	947.476	7
A8	0.612	3	966.776	5
A9	0.459	10	1014.68	2
A10	0.606	4	807.148	10

**Figure 8:**
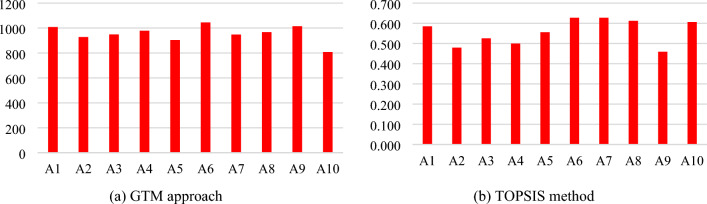
Results comparison.

According to both methods, the same alternative is selected and ranked first. Among the list of assumed alternatives, the A_6_ authentication alternative is considered as best choice or solution in terms of features related to authentication in the IoT environment. The results comparison of proposed methods such as GTM and TOPSIS approach are visually represented in Fig. 8a and b.

**Figure 9:**
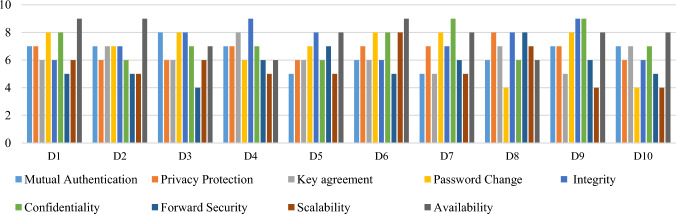
Authentication alternative features input values.

From the results of this research, it is observed that the A_6_ authentication alternative is ranked first among the list of alternatives. The input values provided to the features for the high-ranked alternative (A6) and all the selected authentication alternatives are given in Fig. 9. Among the list of criteria features, the most important features that should be given high preference for designing an authentication scheme in healthcare are password change, availability, confidentiality, privacy protection, and mutual authentication. The suggested assessment framework can be adopted to make rational decisions about the selection of an authentication scheme in real-world situations, especially in the healthcare domain. The results of this study will enable researchers to provide better security by adding more security to the existing authentication schemes.\

As this is the first framework of its type it is necessary to evaluate the process and results by using evaluation methods. Therefore, the framework presented in this study is also tested and verified by using two survey-based methods i.e. evaluation by experts and evaluation by surveying. The complete details about both evaluation methods are given below.

### Features/parameters evaluation

As already mentioned, performed two case studies were performed by consulting security experts. After building this framework, it was essential to evaluate the proposed framework by experts because of its theoretical and newbie nature, particularly in this domain. The proposed authentication evaluation framework is evaluated and tested for accuracy, precision, and recall. Decisions about the selection of relevant, irrelevant, not-recommended, and recommended authentication features are very important to keep the framework working correctly in terms of methodology and results. For this purpose, this framework is validated by an expert group in the field of IoT security. To do so, four variables were taken to denote the classification purpose. The results obtained from the expert group are divided into relevant, irrelevant, recommended, and not-recommended features. Similarly, the number of features suggested by experts and the proposed evaluation framework is represented by "a" and “b” represents the number of evaluations only suggested by the proposed evaluation framework. Features only proposed by the expert panel are represented by "c” and features not proposed by the proposed evaluation framework nor by the expert panel are denoted by "d." This framework is also evaluated by evaluation metrics such as accuracy, precision, and recall by surveying the security expert. The evaluation procedure employed in this research is inspired by the method suggested in^75^ which is used usually for the assessment of context-based recommendation systems. The following Eqs. (6), (7), and (8) are used for obtaining the evaluating parameters.

6 $${\text{Accuracy}} = {\raise0.7ex\hbox{${\left( {{\text{a}} + {\text{d}}} \right)}$} \!\mathord{\left/ {\vphantom {{\left( {{\text{a}} + {\text{d}}} \right)} {\left( {{\text{a}} + {\text{b}} + {\text{c}} + {\text{d}}} \right)}}}\right.\kern-\nulldelimiterspace} \!\lower0.7ex\hbox{${\left( {{\text{a}} + {\text{b}} + {\text{c}} + {\text{d}}} \right)}$}}$$

7 $${\text{Recall}} = {\raise0.7ex\hbox{${\left( {\text{a}} \right)}$} \!\mathord{\left/ {\vphantom {{\left( {\text{a}} \right)} {\left( {{\text{a}} + {\text{c}}} \right)}}}\right.\kern-\nulldelimiterspace} \!\lower0.7ex\hbox{${\left( {{\text{a}} + {\text{c}}} \right)}$}}$$

8 $${\text{Precision}} = {\raise0.7ex\hbox{${\left( {\text{a}} \right)}$} \!\mathord{\left/ {\vphantom {{\left( {\text{a}} \right)} {\left( {{\text{a}} + {\text{b}}} \right)}}}\right.\kern-\nulldelimiterspace} \!\lower0.7ex\hbox{${\left( {{\text{a}} + {\text{b}}} \right)}$}} .$$

The complete details of evaluation parameters obtained from each expert panel in comparison to the proposed evaluation criteria are listed in Table 10.

**Table 10:** Results of recommendation evaluation parameters.

Expert	a	b	c	d	Accuracy (%)	Recall (%)	Precision (%)
1	8	1	1	7	88	89	89
2	16	0	1	15	97	100	94
3	17	1	1	14	94	94	94
4	12	0	1	7	95	100	92
5	13	1	0	12	96	93	100
6	28	2	2	12	91	93	93
7	12	1	0	9	95	92	100
8	9	0	2	16	93	100	82
9	10	0	1	12	96	100	91
10	23	2	1	8	91	92	96
Average					94	95	93

**Figure 10:**
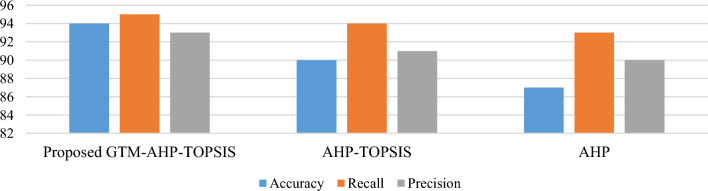
Comparing the proposed method with existing works.

In this research, an assessment model supported by integrated assessment methods is presented. It is compared with the previously applied methods in this area such as AHP and AHP-TOPSIS. As the number of criteria affects the AHP method working procedure and results can be affected. This method attempts to minimize these problems by providing a more sophisticated assessment based on the application of Delphi, GTM, and AHP-TOPSIS methods. The comparison of the features in the proposed model with the other methods is given in Fig. 10. The proposed methodology produces better results for the evaluation metrics used for feature assessment.

### Evaluation by survey

It is also indispensable to evaluate the proposed framework by conducting an expert survey. This survey is conducted with three groups of participants. The participants of this survey belong to the network security domain and are currently pursuing MS and Ph.D. degrees. The number of experts in the first, second, and third groups are respectively 8, 13, and 9. They evaluated the framework based on a 5-point scale. A 5-point scale is used for survey questions, according to scale 5 numeric value represents strongly agreed and 1 indicates strongly disagreed. In this survey, 27 questions are divided into different categories. These categories are security, usability, information knowledge, and effectiveness. The complete procedure of evaluating the suggested evaluation framework by the experts’ groups according to the evaluation metrics given in Table 11.

**Table 11:** Evaluation metrics and feedback from the expert groups.

Evaluation Metrics		Expert groups		
Security		EG-1	EG-2	EG-3
1	This method evaluates the overall security aspects related to authentication	4.3	4	4.5
2	It can be used for all types of authentication methods evaluation employed in a healthcare environment	4	4.2	3.8
3	All the perquisites for authentication are included	4.2	4	4.2
4	It will help in building more secure authentication schemes or methods	4.5	4	4.3
5	It will mitigate the impact of risks in a healthy care environment	4	3.5	4.5
6	It is selecting security solutions in the healthcare domain	4	3.5	3.8
Average		4.1	3.88	4.3
Usability				
7	The proposed evaluation framework is easy to use	4	3.6	4.2
8	It will support all types of authentication methods	4.1	3.7	4.5
9	The assessment procedure carried out by the proposed framework is user-friendly	4.5	4.4	4.3
10	It will provide a flexible approach irrespective of authentication methods	4	3.4	4.1
11	It will help enhance the user experience	4	4.3	4.4
Average		4.1	3.88	4.3
Information and knowledge				
12	The proposed evaluation framework will provide an opportunity to learn more about security	4.1	4.6	4.4
13	The feedback provided related to authentication methods can be used to improve the existing methods employed for authentication in the healthcare environment	3.7	4.2	4.4
14	It assists assisting giving information about the weaknesses of authentication methods	4.2	4.2	4.5
15	This framework is enlightening the end-users to pick the right security solution	4.4	4.2	4.1
Average		4.1	4.3	4.35
Effectiveness				
16	The results yielded by this framework are correct	4.8	4.6	4.7
17	The quantitative results are consistent	4.5	4.5	4.5
18	This framework has rightly incorporated the security issues prevailing in the healthcare environment	4.4	4.6	4.7
19	The most updated and relevant features are included in this study	4.2	3.8	4.3
20	The evaluation framework uses modern techniques	4.5	4.5	4.4
21	The proposed criteria design can be used as a yardstick for the future use	4.2	4.1	3.9
22	The proposed study focuses on addressing the security issues in well-manner	4	4.5	4.1
23	The features are selected from authentic sources	4.5	4	4.5
24	This framework covers the most updated issues in a detailed fashion	4	4.2	3.9
25	The validation mechanism of the framework is properly carried out	4.8	4.5	4.6
26	This framework follows an updated validation method	4.5	4.5	4.4
27	The framework is more effective while upgrading the security of solutions	4	3.7	3.5
Average		4.3	4.2	**4.3**
Accumulative average		4.15	4.06	4.12

This evaluation procedure has made it significantly clear that the average values of all the numbers are above 4. It indicates that the suggested evaluation system has received positive feedback from every expert panel. Positive input has been received from every group member, and they all support the recommendation of this evaluation framework for authentication systems in the healthcare sector due to its effective outcomes and procedure.

## Practical implications

The majority of the existing approaches employed for the decision-making purpose are leveraging the AHP-TOPSIS models however the proposed model uses a novel approach i.e. GTM (AHP-TOPSIS). This model has practical utility in the healthcare sector where sensitive data about the patients are captured and handled. Thus the decision about the most appropriate security algorithms is vital for the security personnel in the healthcare sector. It is This model can be very effective in making the right and informed decision regarding the deployment of secure security protocols to deal with healthcare vulnerabilities. TOPSIS model may recommend the use of adaptive authentication measures and constant monitoring of real-time healthcare data.

The model proposed will help the stakeholders such as network engineers or network administrators to determine the most optimal security solutions for their healthcare security requirements. This model has the potential to evaluate the security alternatives based on ideal point distance by leveraging TOPSIS. Thus this information allows stakeholders to understand why certain solutions are preferred over others. Consequently, the most suitable and informed decisions driven by empirical analysis are made.

TOPSIS offers visualization such that the relative weights of criteria are depicted visually. This visualization makes it easier for decision-makers to grasp the significance of each criterion and helps in understanding the overall evaluation process.

TOPSIS offers sensitivity analysis which is very helpful for the decision-makers in the healthcare sector to check the robustness and efficiency of the recommended model. The model changes its results according to the criteria weights or importance. Thus it helps the healthcare decision-maker to get a full understanding of the uncertain rendering in the evaluation model.

As the people working in the healthcare sector have very little technical knowledge and training experience about network security awareness this model driven by GTM (AHP-TOPSIS) can be more effective in evaluating the effectiveness of security algorithms to be employed.

## Conclusion and future work

The security of IoT devices has always been a major concern, especially in the healthcare domain. To address the security issues of IoT devices, many authentication schemes are presented. The selective installation of the right authentication scheme to meet the security requirements remains an open issue. Therefore, in this research work, the prime focus is to identify and choose the most ideal choice of authentication solution/scheme for IoT devices based on the features of authentication. For this purpose, a feature-based authentication framework is presented by using the GTM approach in an IoHT-based system. The objective is to deploy the right security solutions for IoT devices by looking into the most indispensable features required for the authentication of any device. In the first phase of the IoHT assessment network, features are selected from the literature based on their commonality and frequency of occurring in the literature study. After setting the benchmark, a case study is conducted to get all the required information, which is then classified into different authentication features. Then, the IoHT authentication assessment framework is presented that makes decisions related to the selection of the best authentication solution for IoHT devices among the list of alternatives. This assessment framework uses the GTM approach for the selection of the best solution in terms of the degree of security by using authentication features as a benchmark. This method is based on a mathematical approach that evaluates and installs the most appropriate authentication scheme as an alternative in terms of its features. The results obtained from this approach are further justified by using the AHP-TOPSIS method. The TOPSIS method validates that the quantitative results of the proposed evaluation framework are accurate and consistent.

Some of the major limitations of this study are as:

One of the limitations related to the study is that the proposed evaluation framework is merely taking into account the security aspect of authentication schemes in the healthcare field. It does not consider the energy, authentication time, complexity of the algorithm, memory space, key size, or latency issues. The criteria-designing procedure has originated from literature and expert interviews. Some of the important features can likely be skipped. The features suggested by the expert panel can also be a concern as the criteria are not absolute, it is relative. Similarly, the data collection procedure has been significantly affected by the experts' opinions during the case study. The decision matrix can be the one potential solution to resolve this issue of subjectivity and biases.

Similarly, during the framework validation and testing process, the AHP method can be less efficient especially when the number of criteria features and alternatives increases. This issue can be resolved by applying Fuzzy or Gaussian methods with AHP or more advanced methods. The linguistic model is also another addition to get more desirable outcomes.

All the integrated methods follow different working procedures for the evaluation AHP rely upon the hierarchical relationship among criteria and alternatives, GTM provides graphic and matrix representations of real-world problems and TOPSIS uses the ideal solution for prioritizing alternatives for given criteria. This integration creates a more complex model with a higher level of abstraction. Sometimes, it becomes so difficult for stakeholders to understand the decision-making procedure and outcomes driven by the combination of these methods.

In future work, we are looking forward to addressing all the existing complexities and limitations by designing a more intelligent and efficient decision-making model for the evaluation and ranking of authentication solutions based on enhanced evaluation criteria.

## Data Availability

All the data analyzed or used in this research study are displayed in the manuscript file.
